# Effect of vitamins A/C primed human gingival mesenchymal stem/progenitor cells in preventing medication-related osteonecrosis of extraction socket: an animal study

**DOI:** 10.1007/s10266-025-01288-x

**Published:** 2026-01-04

**Authors:** Fatma I. Elfaiedi, Nahed S. Korany, Karim Fawzy El-Sayed, Nermeen AbuBakr

**Affiliations:** 1 Department of Oral Biology, Faculty of Dentistry, Cairo University, Cairo, Egypt; 2 Department of Oral Medicine and Periodontology, Faculty of Dentistry, Cairo University, Cairo, Egypt; 3 Clinic for Conservative Dentistry and Periodontology, School of Dental Medicine, Christian Albrechts University, Kiel, Germany; 4 Stem Cells and Tissue Engineering Research Unit, Faculty of Dentistry, Cairo University, Cairo, Egypt

**Keywords:** Ascorbic acid, Vitamin A, Wound healing, Osteonecrosis, Stem cells

## Abstract

Human gingival mesenchymal stem/progenitor cells (hGMSCs) exert remarkable tissue regeneration properties. A combination of vitamins C and A at specified concentrations enhanced cellular dedifferentiation and multipotency of hGMSCs. To our knowledge, vitamins A/C-primed hGMSCs in the prevention of medication-related osteonecrosis of the jaw (MRONJ) have not been investigated. Thus, this study evaluated the impact of vitamins A/C in pluripotency-inductive concentrations on the regenerative/reparative potential of hGMSCs in preventing MRONJ development in rats. Thirty albino rats (*n* = 10/group) were administered zoledronic acid and dexamethasone three times a week for 4 weeks. The upper first molar was extracted 2 weeks following the first administration. Immediately after extraction, rats received normal saline (group Ι), hGMSCs (group ΙΙ), and hGMSCs primed with vitamins A/C (group ΙΙΙ). Rats were euthanized 2 and 4 weeks after extraction. The maxillae were analyzed using dual-energy X-ray absorptiometry, histologically, histomorphometrically, and by quantitative real-time polymerase chain reaction analysis. Groups II and III displayed elevated bone mineral density, with a significant rise in group ΙΙΙ relative to group ΙΙ at 4 weeks. Histomorphometrically, groups II and III revealed an elevation in area percentage of newly generated bone, with a significant rise in group ΙΙΙ at both time intervals. Gene expression of transforming growth factor-β1 (TGF-β1) and runt-related transcription factor 2 (Runx2) presented a significant elevation in groups II and III, with a significant rise in group ΙΙΙ at both time intervals. In conclusion, vitamins A/C-primed hGMSCs at the tested pluripotency-inductive concentrations prevented MRONJ development, primarily through encouraging wound healing and bone repair in the extraction sockets of rats.

**Supplementary Information:**

The online version contains supplementary material available at https://doi.org/10.1007/s10266-025-01288-x.

## Introduction

Medication-related osteonecrosis of the jaw (MRONJ) is a multifactorial condition, in which an infection, following bone injury (e.g., tooth extraction), combined with repressed bone remodeling caused by antiresorptive medications (e.g., bisphosphonates (BPs)), can result in persistently exposed bone without epithelial coverage accompanied by pain, infection, and sinus formation. The resultant clinically challenging situation could markedly reduce the patient’s life quality [1].

To date, although different therapeutic approaches were advocated, no consensus exists for a widely accepted effective strategy for the prevention/treatment of MRONJ. Among the strategies examined, mesenchymal stem/progenitor cells (MSCs)-based tissue engineering was reported to hold a promising potential in this context [2]. Human gingival mesenchymal stem/progenitor cells (hGMSCs), adult stem/progenitor cells, which can be easily separated from the gingiva, are marked by multipotent differentiation abilities, remarkable proliferation and regeneration capacities [3, 4], as well as immunomodulatory properties [5–7]. Previous studies have shown that hGMSCs can be effectively combined with bioactive regenerative biomaterials and dentinal derivatives. These combinations supported cell viability, matrix deposition, and osteogenic differentiation, highlighting their promising role in bone and soft tissue regeneration [8, 9].

Ascorbic acid (vitamin C) is a strong antioxidant, necessary for collagen generation and cellular growth and activity [10, 11]. It exerts anti-inflammatory activities, can promote immune defense as well as wound healing events [12], and encourages the growth of mesenchymal cells into osteoblasts [13]. An in vitro investigation demonstrated that ascorbic acid at a certain concentration enhances hGMSCs’ stemness, proliferation, and differentiation properties [14].

Likewise, fat-soluble vitamin A, an important element in the adaptive immune system, is a cofactor for the development and distinction of regulatory T cells [15] and is crucial for the osteogenic differentiation of hGMSCs [16] and regeneration [17]. Importantly, retinoic acid signaling pathways were suggested as therapeutic targets in bone diseases [18].

Strikingly, earlier studies demonstrated that a combination of vitamins C and A at specific doses impacts cellular epigenetics by demethylating nuclear bases, which causes adult cells to dedifferentiate into pluripotent ones [19, 20]. A combination of vitamin C and A at these pluripotency-inductive concentrations with hGMSCs could foster their therapeutic effect in the early control/prevention of MRONJ, and to our knowledge, it has not been investigated. Thus, this animal research intended to investigate the effect of vitamins A/C-primed hGMSCs in the early control/prevention of medication-related osteonecrosis in extraction sockets of albino rats.

## Materials and methods

### Isolation of hGMSCs

Gingival tissues were obtained from healthy partially impacted third molars that were surgically extracted with free gingival soft tissue collars. The gingival tissues were submerged in phosphate buffer solution complemented with 100 µg/ml streptomycin, 1% amphotericin, and 100 U/ml penicillin at 4 °C for a period not exceeding 24 h. Tissues were deepithelialized and divided into little pieces, washed with minimum essential medium (MEM), and antibiotics (100 µg/ml streptomycin, 100 U/ml penicillin). The specimens were put into dry tissue culture flasks for 30 min for adherence. The basic medium included MEM complemented with 400 mmol/ml L-glutamine, 15% fetal calf serum, 100 µg/ml streptomycin, and 100 U/ml penicillin. Cells were allowed to proliferate after the flasks were incubated at 37 °C with 5% CO_2_. Phase contrast inverted microscopy was utilized to inspect the culture flasks, and the basic medium was replaced three times every week. Cells were separated using 0.10% trypsin–EDTA and counted once they had reached 80–85% confluence. Trypan Blue was used to verify their vitality to be finally cultured at a density of 30 cells/cm^2^ in culture flasks. The flasks were then incubated at 37 °C with 5% CO_2_ [21].

### Identification of hGMSCs

#### Flow cytometric analysis

Upon reaching confluence, third passage hGMSCs were characterized by flow cytometry. Primary antibodies against human CD34, CD44 (eBioscience, Waltham, USA), CD45, CD73, CD90, and CD105 (Miltenyi Biotec, Bergisch Gladbach, Germany) were utilized. Cells were stained with fluorescein isothiocyanate (FITC) or phycoerythrin (PE)-conjugated primary antibodies at room temperature. PE- or FITC-conjugated human IgG were employed as isotype controls. A CytoFLEX flow cytometer and CytExpert Software (Beckman Coulter, USA) were used for the analyses.

#### Multilineage differentiation assays

Osteogenic media was used to cultivate third passage hGMSCs. To identify calcified deposits, cell cultures were stained with Alizarin Red on day 14. hGMSCs were cultivated in adipogenic inductive media for 21 days to determine their capacity for adipogenic differentiation. Oil-Red-O staining was utilized to assess the existence of lipid drops. Chondrogenic inductive media was used to incubate hGMSCs to examine their capacity for chondrogenic differentiation. At day 35, chondrogenic differentiation was assessed by staining glycosaminoglycans with Alcian Blue. Basic medium was used for the same durations as the respective controls. Three times per week, all media were renewed [22].

### Experimental study design

#### Sample size calculation

Using a 0.05 alpha threshold, a statistical power of 80%, and a 1.13 effect size (Cohen’s f) determined from a previous investigation [23], a total of 18 animals were required (*n* = 18). The sample size was raised to enhance the robustness of the statistical analysis to be 30 samples in total, with 10 samples in each group and 5 samples in each subgroup. R statistical analysis software version 4.5.0 for Windows was utilized to calculate the sample size.

#### Animals

Thirty (*n* = 30) healthy adult 7-weeks-old male albino rats, weighing about 195 ± 20g were employed. The animals were kept in a pathogen-free environment with a humidity of 50 ± 1%, a temperature of 22 ± 1 °C, and a 12-h light/dark cycle and were fed normal chow and water.

#### Induction of MRONJ-like animal model

Three times a week for 4 weeks, zoledronic acid (ZA) (66 µg/kg) (Novartis, Basel, Switzerland) and dexamethasone (5 mg/kg) (Amriya Pharmaceuticals, Alexandria, Egypt) were injected subcutaneously to induce osteonecrosis. Two weeks following the first administration, all rats were subjected to maxillary left first molar atraumatic extraction [24]. The rats were anesthetized with an intraperitoneal injection of a mixture of ketamine (70 mg/kg; Ketamar^®^ 5%, Amoun Pharmaceutical Co., Qalyubia, Egypt) and xylazine (10 mg/kg; Xyla-Ject^®^ 2%, ADWIA Co., Cairo, Egypt). Each rat was then positioned with the head gently tilted backward, and the mouth was carefully opened while the tongue was depressed. The molar was grasped with a curved mosquito hemostat and gently luxated with slow bucco-palatal movements until loosening was achieved, followed by gradual buccal traction to remove the tooth with intact roots. Rats were fed a soft diet for 1 week after extraction to decrease trauma from mastication and hard food. ZA and dexamethasone injections were continued for 4 weeks. Subcutaneous injection of buprenorphine (0.05 mg/kg) was administered every 12 h for 48 h following extraction for pain relief [25].

#### Groups

Immediately after extraction, rats were split randomly into three groups (www.random.org, *n* = 10/group): (1) group I: was administered normal saline in the extraction site; (2) group II (hGMSCs group): received hGMSCs (1 × 10^6^ cells/mL) [26] in the extraction site; (3) group III (vitamins A/C-primed hGMSCs group): received hGMSCs (1 × 10^6^ cells/mL) with vitamin C 250 μmol/L [20] and vitamin A 20 μmol/L [18] in the extraction site. In each group, either saline solution or hGMSCs were loaded onto a sterile absorbable hemostatic gelatine sponge [27] (Cutanplast^®^ Standard, Mascia Brunelli S.p.A, Milano, Italy), trimmed to approximately 2 × 3 × 2 mm. The sponge was gently compressed and placed into the extraction socket. It was left in situ and gradually resorbed within the socket.

#### Blinding

For each animal, four investigators were involved. The allocation to the experimental group was only known to the first investigator. The second investigator was the only one to evaluate dosage delivery for every animal during the trial. Without being aware of the groups, the third investigator evaluated the results in a masked manner. The experimental groups were unknown to the statistician who conducted the data analysis.

### Animal euthanasia and tissue preparation

Rats from each group (*n* = 5/time point) were euthanized using anesthesia overdose 2 and 4 weeks post-extraction. All animals survived to the study end points, with no losses. The maxillae were collected and subjected to the following analyses.

#### Bone mineral density (BMD) analysis

BMD (primary outcome) of the collected maxillae was examined by dual-energy X-ray absorptiometry (DEXA) (MEDIX DR, Medilink, Mauguio, France) to determine hard tissue repair in teeth extraction sockets (region of interest). The scanning speed was 60 mm/s, and the resolution was 0.5 × 0.5 mm. Results were displayed automatically in g/cm^2^ [22].

#### Histopathologic examination

All maxillae, including the extraction sockets, were stained with hematoxylin and eosin (H&E) in accordance with standard protocols for histological interpretation by a light microscope [28].

#### Histomorphometric analysis

Leica QWin 500 software (Leica Microsystems, Heerbrugg, Switzerland) was used to evaluate the area percentage of the newly created bone in the extraction socket location. For every specimen, ten fields were measured at a total magnification of 200 × . A standard measuring frame with an area of 118,476.6 µm^2^ was used to compute the bone area percentage [22].

#### Quantitative real-time polymerase chain reaction (qRT-PCR) examination

qRT-PCR was employed to identify the mRNA expression of transforming growth factor-β1 (TGF-β1) and runt-related transcription factor 2 (Runx2) in bone and mucosal tissues of the extraction socket. β-Actin was employed as a housekeeping gene. Total RNA was isolated from homogenized tissue specimens using a Qiagen RNeasy mini kit. Complementary DNA (cDNA) was synthesized from the total RNA of each sample using a high-capacity cDNA reverse transcription kit. The resultant cDNA was then amplified on a 48-well plate utilizing a StepOne^™^ System with software version 2.3 (Applied Biosystems, Waltham, Massachusetts, USA). The thermocycling profile involved 15 min of enzyme activation at 95 °C, followed by 40 cycles of 60 s at 95 °C for denaturation, 60 s at 60 °C for annealing, and 60 s at 72 °C for extension [29]. Primer sequences of the genes are listed in Table 1.

**Table 1:** Primer sequences of the studied genes

Gene	Forward	Reverse	Accession number
TGF-β1	5′-CCTGGAAAGGGCTCAACAC-3′	5′-CTGCCGTACACAGCAGTTCT-3′	X52498
Runx2	5′-TCCCAGTATGAGAGTAGGTGTCC-3′	5′-GGCTCAGATAAGAGGGGTAAGAC-3′	NM_001278483
β-actin	5′-CTATGTTGCCCTAGACTTCG-3′	5′-AGGTCTTTACGGATGTCAAC-3′	NM_031144.3

### Statistical analysis

Microsoft Excel 2016, GraphPadPrism, and the Statistical Package for Social Sciences computer program version 20 (SPSS 20) were used to conduct the statistical analysis. Shapiro–Wilk and Kolmogorov–Smirnov tests were used to explore data for normality, and the results revealed that all of the data were normally distributed. Mean and standard deviation were utilized to display all quantitative data. A paired t test was utilized to compare measures taken 2 and 4 weeks apart, and the one-way ANOVA test and Tukey post hoc test for multiple comparisons were utilized to compare groups. A significant threshold of *P* < 0.05 was established.

## Results

### Phenotypic identification of hGMSCs

**Fig. 1:**
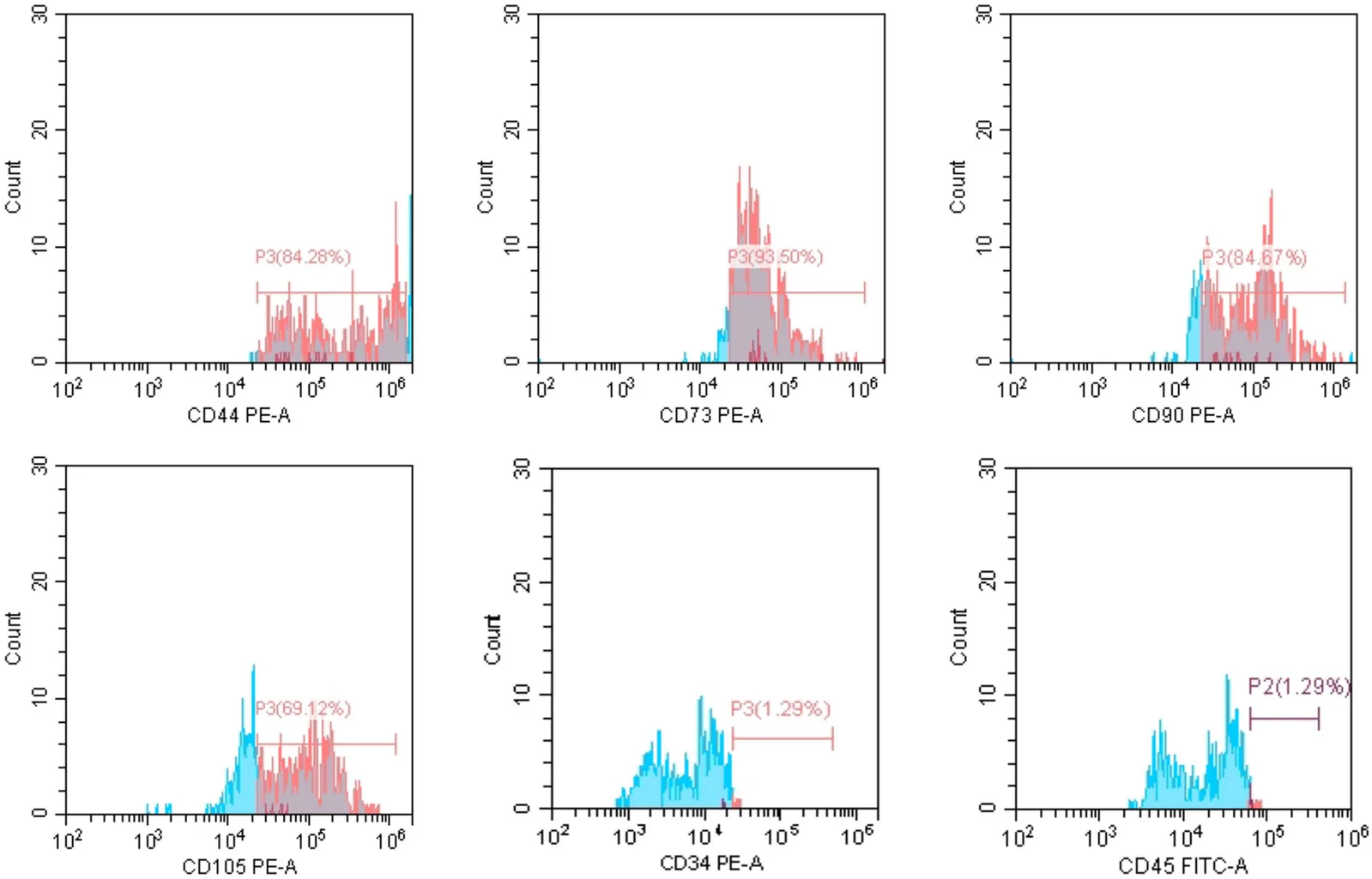
Flow cytometric analysis of cell surface marker expression showing that hGMSCs were positive for MSCs markers CD44, CD73, CD90, and CD105, while negative for hematopoietic markers CD34 and CD45

Flow cytometric analysis of cell surface markers showed positivity for CD44 (84.28%), CD73 (93.50%), CD90 (84.67%), and CD105 (69.12%) and negativity for CD34 (1.29%) and CD45 (1.29%) (Fig. 1).

### Morphological identification of hGMSCs

**Fig. 2:**
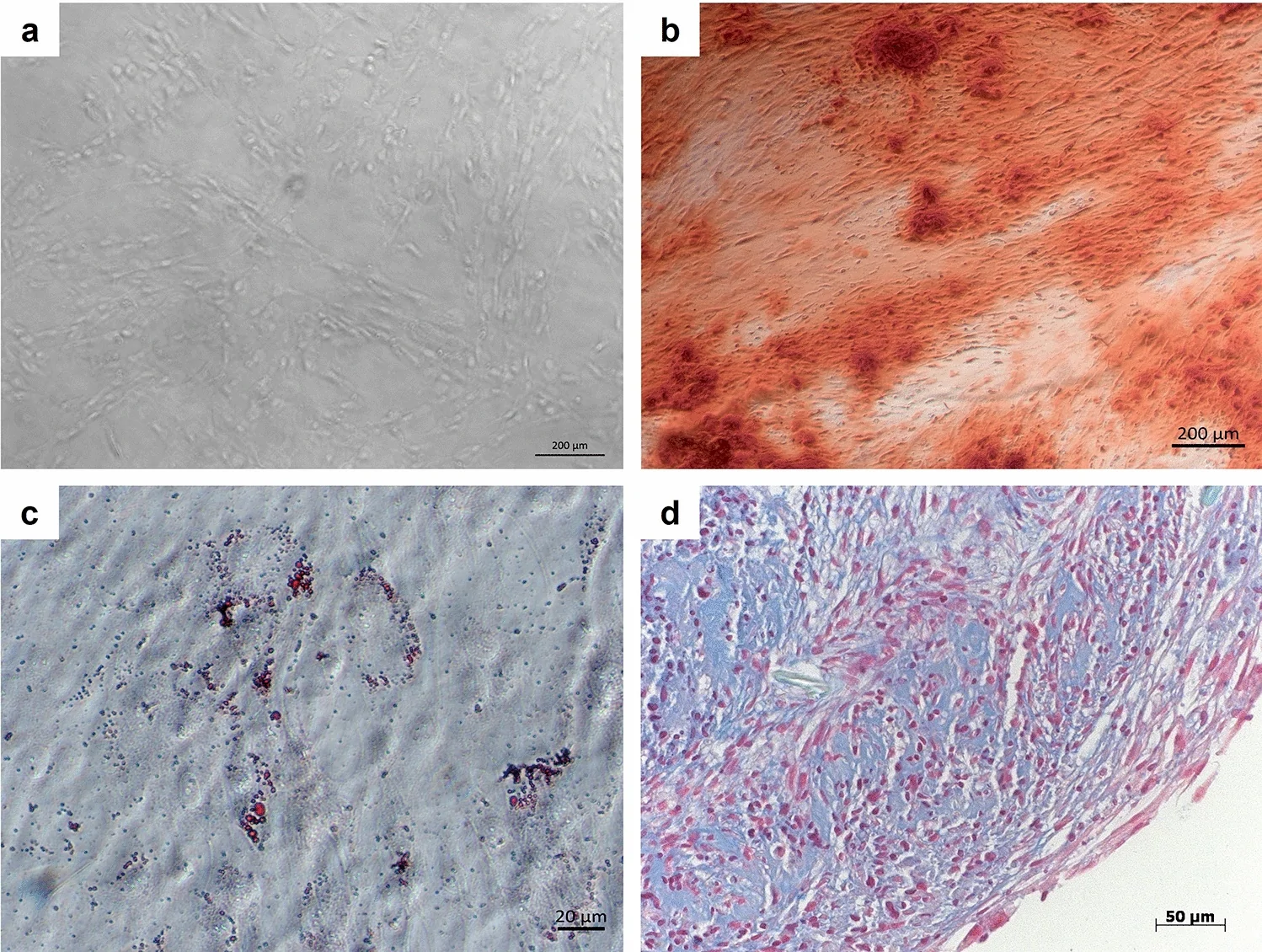
A photomicrograph showing **a** Phase contrast microscopic appearance of hGMSCs, **b** Alizarin Red staining of the osteogenically stimulated hGMSCs, **c** Oil-Red-O staining of the adipogenically stimulated hGMSCs, **d** Alcian Blue staining of the chondrogenically stimulated hGMSCs

At the third passage, hGMSCs exhibited a fibroblast-like morphology (Fig. 2a). Alizarin red-positive calcified deposits formed as a sign of the osteogenic differentiation of hGMSCs (Fig. 2b). Intracellular presence of Oil-Red-O positive lipid drops indicated adipogenic differentiation (Fig. 2c). The synthesis of glycosaminoglycans that stained favorably with Alcian Blue indicated chondrogenic differentiation (Fig. 2d).

### Bone mineral density

**Fig. 3:**
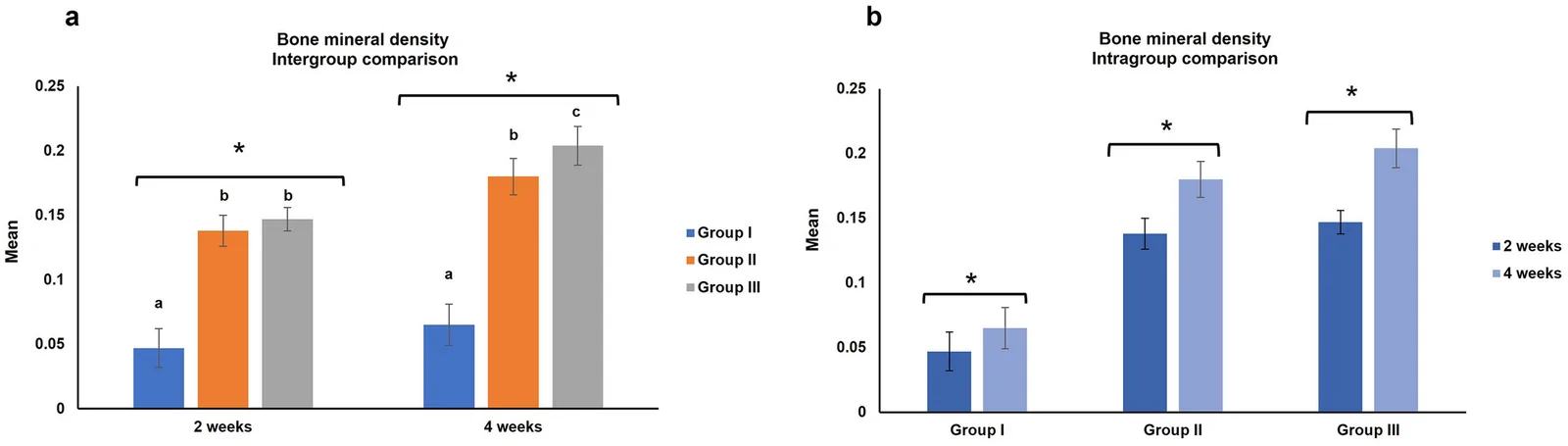
Bar chart showing bone mineral density in all groups after 2 and 4 weeks (**a**) intergroup comparison, * indicates a significant difference between groups using ANOVA. Bars with different letters indicate a significant difference using Tukey post hoc test, and (**b**) intragroup comparison, * indicates a significant difference between groups using the paired t test. Significance at *P* < 0.05. *n* = 5/time point

Two and four weeks post-extraction, a significant difference in BMD was notable between the groups (*p* = 0.0001). Group Ι showed the lowest BMD, while groups II and III showed a significant increase in BMD compared to group Ι, with insignificant differences between them at 2 weeks. However, there was a significant variation between groups II and III, in favor of group III, at 4 weeks (Fig. 3a; Supplementary Table S1). In addition, quantified data of DEXA showed a significant rise in BMD from 2 to 4 weeks in all groups (Fig. 3b; Supplementary Table S2).

### Histological results

**Fig. 4:**
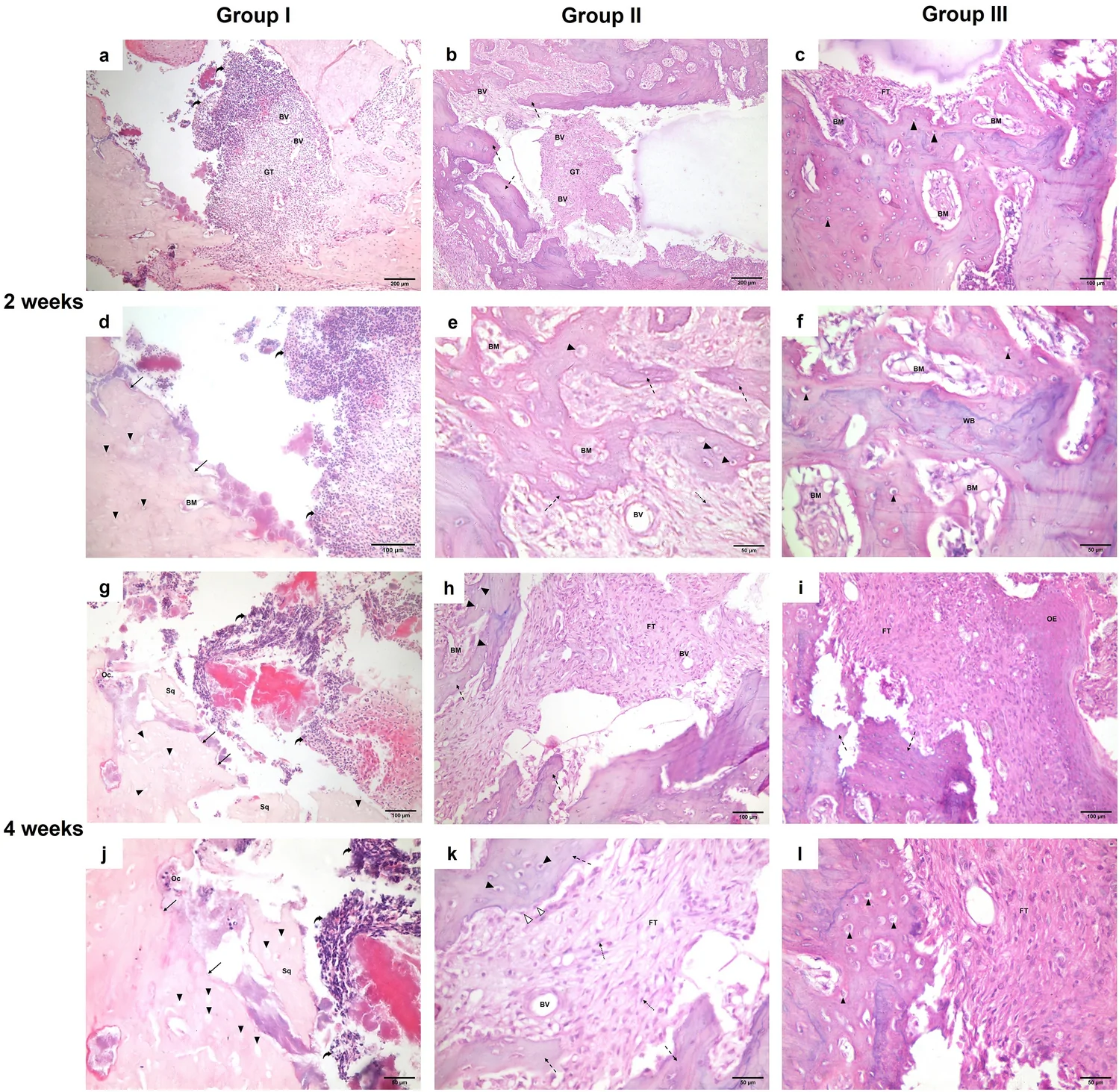
Representative H&E-stained photomicrograph of tooth extraction sockets of groups I, II, and III at 2 weeks (**a–f**) and 4 weeks (**g–l**). Group I (**a**, **d**, **g**, **j**) showing granulation tissue (GT), blood vessels (BV), inflammatory cell infiltrate (curved arrows), scalloped borders (arrows), empty lacunae (black arrowheads), empty marrow spaces (BM), sequestrate (Sq), and osteoclasts (Oc). Group II (**b**, **e**, **h**, **k**) showing granulation tissue (GT), blood vessels (BV), marrow spaces (BM), bone trabeculae (dashed arrows), fibroblasts (dotted arrows), fibrous tissues (FT), osteocytes (black arrowheads), and osteoblasts (white arrowheads). Group III (**c**, **f**, **i**, **l**) showing marrow spaces (BM), osteocytes (black arrow heads), bone trabeculae (dashed arrows), woven bone (WB), oral epithelium (OE), and fibrous tissues (FT)

Histologically, group Ι showed necrotic bone with empty osteocytic lacunae, empty marrow spaces, and uneven resorption of the bone surface. The extraction socket was filled with dense inflammatory cell infiltrates and numerous blood vessels (Fig. 4a, d). After 4 weeks, a higher amount of necrotic bone, more empty lacunae, and bone sequestrate with scalloped borders were visible in the socket. The socket cavity revealed intense inflammatory cell infiltration with a decreased number of blood vessels (Fig. 4g, j).

Group II at 2 weeks revealed that the newly formed bone was sparse and dispersed, enclosing randomly distributed large-sized viable osteocytes and fibrocellular marrow spaces. The new bone was restricted mainly to the internal surfaces of the alveolar socket with predominant osteoblastic activity. The alveolus was filled with granulation tissue, which contained more dilated blood vessels, more fibroblasts, and fewer inflammatory cells (Fig. 4b, e). At 4 weeks, the socket cavity contained larger, more interconnected bone trabeculae that were lined with osteoblasts and enclosing plumbed osteocytes. Fibrous tissue enclosing an increased number of dilated blood vessels and mature collagen fiber bundles was observed filling most of the socket (Fig. 4h, k).

In group III, at 2 weeks, there was newly created woven bone lining the socket basally and laterally, enclosing plumbed osteocytes and fibrocellular marrow cavities. The entire core region of the socket was filled with fibrous tissue as mature collagen fibers intermingled with immature ones (Fig. 4c, f). At 4 weeks, there was clear evidence of bone healing, and the socket was completely filled with more organized tissue. At the coronal end of the socket, there was fibrous tissue that surrounded mature collagen fiber bundles that were covered with an oral epithelial layer. Large, interconnected mature bone trabeculae enclosing plumbed osteocytes filled the socket (Fig. 4i, l).

### Histomorphometric results

**Fig. 5:**
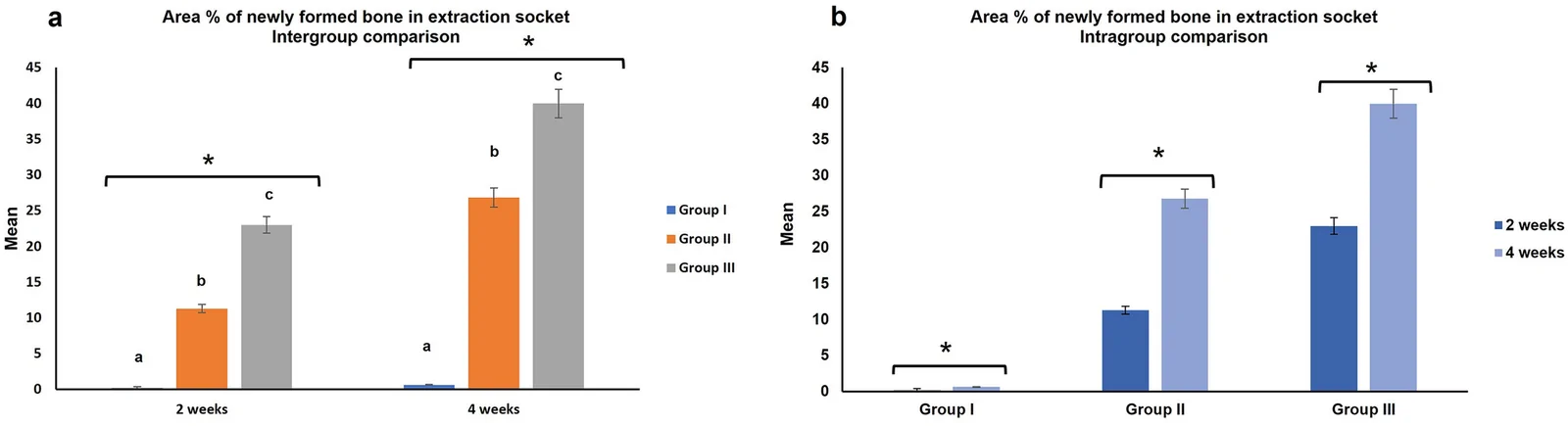
Bar chart showing the area percentage of newly formed bone in the extraction socket of all groups after 2 and 4 weeks (**a**) intergroup comparison, * indicates a significant difference between groups using ANOVA. Bars with different letters indicate a significant difference using Tukey post hoc test, and (**b**) intragroup comparison, * indicates a significant difference between groups using the paired t test. Significance at *P* < 0.05. *n* = 5/time point

After 2 and 4 weeks of extraction, there was a significant variation in the area percentage of newly formed bone among all groups (*p* = 0.0001). Group Ι showed the least area percentage of newly created bone, while groups II and III showed a rise in the area percentage of newly formed bone compared to group Ι. Additionally, there was a significant variation between groups II and III, in favor of group III, at both time points (Fig. 5a; Supplementary Table S3). In the same context, there was a significant rise in the quantity of newly created bone in the extraction socket of all groups from 2 to 4 weeks (Fig. 5b; Supplementary Table S4).

### Quantitative real-time polymerase chain reaction (qRT-PCR) results

**Fig. 6:**
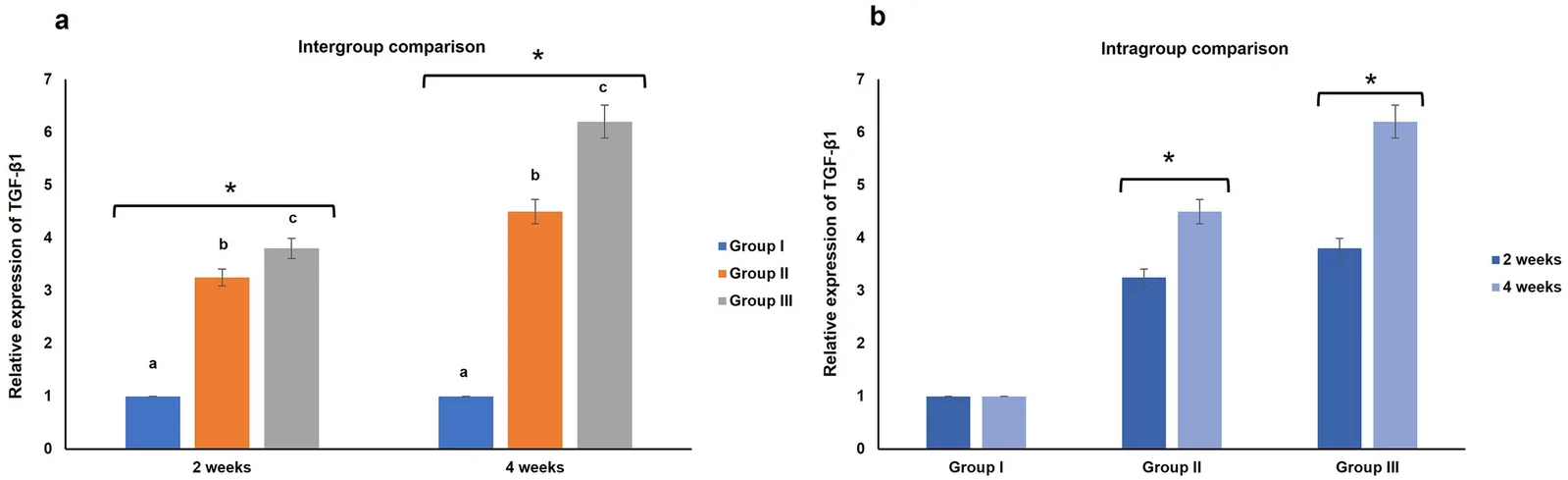
Bar chart showing mRNA gene expression of TGFβ-1 in all groups after 2 and 4 weeks (**a**) intergroup comparison, * indicates a significant difference between groups using ANOVA. Bars with different letters indicate a significant difference using Tukey post hoc test, and (**b**) intragroup comparison, * indicates a significant difference between groups using the paired t test. Significance at *P* < 0.05. *n* = 5/time point

**Fig. 7:**
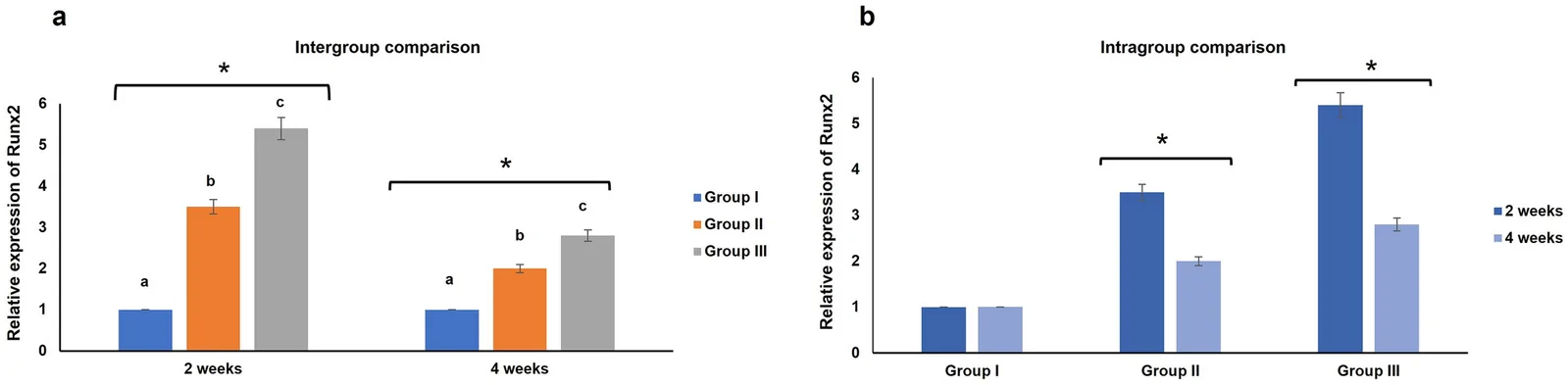
Bar chart showing mRNA gene expression of Runx2 in all groups after 2 and 4 weeks (**a**) intergroup comparison, * indicates a significant difference between groups using ANOVA. Bars with different letters indicate a significant difference using Tukey post hoc test, and (**b**) intragroup comparison, * indicates a significant difference between groups using the paired t test. Significance at P < 0.05. *n* = 5/time point

There was a significant variation in TGF-β1 and Runx2 expressions in groups II and III relative to group I (*p* = 0.0001), with a further significant difference between groups II and III, in favor of group III, at 2 and 4 weeks post-extraction. Intragroup comparisons revealed a significant elevation in TGF-β1 gene expression from 2 to 4 weeks in groups ΙΙ and ΙΙΙ. On the contrary, a significant decline in Runx2 gene expression was noticed from 2 to 4 weeks in groups ΙΙ and ΙΙΙ (Figs. 6 and 7; Supplementary Tables S5–S8).

## Discussion

Since the first report on MRONJ was released [30], various modalities aimed at preventing or treating this life-threatening morbidity have been investigated [31, 32]. The objective of the present study was to evaluate the reparative/regenerative ability of vitamins A/C-primed hGMSCs at pluripotency-inductive concentrations in the prevention of MRONJ in an osteonecrotic rat model.

In line with earlier research [33], ZA was used in combination with dexamethasone to induce osteonecrosis. The maxilla was selected as the evaluation site in this investigation, as it presents a lower risk of complications compared to the mandible. Extractions in the mandible are often associated with fractures and feeding difficulties, whereas the maxilla presents a safer alternative. Its thinner cortical bone and greater vascularity favor better healing outcomes and provide a greater consistency in the subsequent assessment. This methodological choice was also supported by previous studies that have successfully induced MRONJ in the maxilla [34, 35].

hGMSCs were chosen, being readily available and possessing superior biological characteristics [3], including fostered bone repair [5] and a notable capacity for repair/regeneration [7, 36]. A combination of vitamins A and C at particular concentrations would affect cellular epigenetics, via demethylation of nuclear bases, with a subsequent induction of hGMSCs’ stemness and reparative/regenerative capacity [19, 20]. Although this combined application demonstrated remarkable effects on hGMSCs in vitro [37], a combination of both vitamins was not utilized before in an animal model for the prevention of MRONJ.

In the herein study, all interventions were administered immediately after extraction and delivered locally since local administration, as opposed to a systemic one, permits the agent to act on the target without its bioavailability being impacted by digestion and gastrointestinal absorption. Moreover, as it can be administered when the tooth is being extracted, a subsequent translational clinical practice implementation would be straightforward [35].

No graft rejection was observed in the present work, likely due to the combined effects of hGMSCs and dexamethasone. hGMSCs possess low immunogenicity and actively release anti-inflammatory factors that reduce host immune responses [38, 39], while dexamethasone provides additional immunosuppression [40]. Together, these factors could have created a favorable environment that prevented rejection of the transplanted cells during the 2–4 weeks experimental period. This was in line with previous studies that also reported the successful use of hGMSCs in animal models for regenerative approaches [36, 41].

In this investigation, rats were euthanized at 2 and 4 weeks following extraction. This 2–4 weeks observation period was chosen because rodents exhibit faster bone turnover and healing than humans, so this time frame would analogously represent the early and intermediate phases of healing, during which MRONJ-like lesions can be reliably induced in rodents, and is consistent with previous preclinical MRONJ studies [24, 33, 34].

In the present study, DEXA results exhibited a significant elevation in BMD in groups II and III with a significant rise in group III at 4 weeks. These findings were similar to earlier findings where after 4 and 8 weeks of MSC injection into the tail vein of ovariectomy mice (in a cell homing approach), a remarkable rise in BMD was observed [42]. Comparably, the tibias of ovariectomized rats receiving vitamin C therapy recovered their BMD [43]. In the same context, it was reported that a greater BMD was linked to increased vitamin A consumption through diet [44].

In the current work, the histopathological examination of group I at 2 weeks displayed clear osteonecrotic signs, which were aggravated at 4 weeks, verifying the osteonecrosis model employed. The resultant condition imitated the pathology of MRONJ‐like lesions 2 and 4 weeks post-extraction [24] and could be attributed to the fact that administering ZA may hinder tissue healing and regeneration through preventing stem/progenitor cells that are located around the jawbone from migrating to the necrotic area [45]. Moreover, BPs cause blockage in the signaling pathway of bone marrow cells and inhibit the deposition of bone mineral of osteoblasts and bone remodeling in the extraction socket [46]. Additionally, BPs are directly harmful to the oral epithelium, preventing the overlying soft tissue from healing [47].

In group II, many of the histopathologic events linked to osteonecrosis were significantly reversed by hGMSCs at 2 weeks, with more healing signs observed at 4 weeks. The results were concomitant with previous findings, in which MSCs injected into the osteonecrotic femoral head of the rabbit model achieved greater repair, with large quantities of fibrous tissue and cartilage covering the necrotic femoral head [48]. In group III, the onset of MRONJ was suppressed at 2 weeks, with new mature bone formation at 4 weeks, in accordance with earlier findings [49], demonstrating that the local use of an osteogenic inducer improved extraction socket healing in rabbits, enhancing the quality of the created bone, with substantial production of lamellar bone in the sockets. Moreover, all-trans retinoic acid (ATRA) was shown to potentiate bone morphogenic protein (BMP) 9-induced osteogenic differentiation of the mesenchymal progenitor cell line by inducing alkaline phosphatase (ALP) activity in vitro [50]. The histomorphometric analysis of new bone formation further supported these histopathological results. In line with earlier results [51, 52], the area percentage of newly created bone exhibited a significant increase in groups II and III compared to group Ι, with a significant rise in group III at both time intervals.

In accordance with earlier findings observed with adipose-derived stem cells injected in animals receiving BPs [53], in the current work, a significant elevation in TGF-β1 in groups II and III relative to group I, with a significant increase in group III at both time intervals, was further evident. Similarly, mice supplemented with ascorbic acid had significantly elevated TGF-β expression compared to deficient mice after 7 days of wound healing [54]. Moreover, a study on necrotizing enterocolitis found that vitamin A administration activated TGF-β1, which contributed to accelerated healing [55].

In the herein study, the expression of Runx2 in both groups II and III showed a significant rise relative to group I. These findings were consistent with a previous study where ascorbic acid supplementation in hGMSCs cultures was linked to osteogenesis, leading to enhanced mineralization and elevation of osteogenic genes Runx2, BMP-2/4, and osteopontin (OPN) [56]. Furthermore, it was demonstrated that osteoblast differentiation was greatly accelerated by low dosages of vitamin C, as was evident by the increase of Runx2 gene expression [57]. Treatment with retinoic acid was additionally shown to significantly increase Runx2 expression and osteoblastic differentiation [58]. ATRA encouraged bone marrow MSCs to differentiate into osteoblasts and increased Runx2 expression, bone formation, and mineralization density [59]. Yet, being an early osteogenic marker and concomitant with earlier findings [48], Runx2 expression in both hGMSCs groups significantly decreased at 4 weeks compared to 2 weeks.

The pronounced preventive potential observed in vitamins A/C-primed hGMSCs group against MRONJ could be attributed to the complementary roles of retinol and ascorbate in regulating the epigenetic and osteogenic pathways. Retinol and ascorbate act in combination to promote DNA demethylation and enhance reprogramming toward naïve pluripotency by targeting ten–eleven translocation (TET) enzymes. Retinol upregulates TET2 and TET3 expression, while ascorbate sustains their catalytic activity by maintaining iron in its reduced Fe^2^⁺ state. Through these mechanisms, both vitamins facilitate the removal of epigenetic memory and support stem cell renewal [60]. In addition, vitamin C has been shown to potentiate osteoblastic function via enhancing osteoblast proliferation, increasing trabecular bone formation, and inducing bone matrix genes such as ALP, OPN, and Runx2 [61, 62]. Analogously, vitamin A contributes to bone physiology regulation through retinoic acid receptor-mediated signaling [63], with ATRA markedly elevating osteoblastogenesis and osteogenesis [59].

## Limitations

Yet, the current findings should be carefully interpreted in light of the present animal study’s limitations. First, the 2- to 4-week observation period, while appropriate for capturing early and intermediate phases of post-extraction healing in rodents, does not fully replicate the chronic progression of MRONJ in humans, which typically develops over several months. Second, although our findings demonstrate the beneficial effects of primed vitamins A and C in combination with hGMSCs, the exact underlying molecular mechanisms remain unclear. Third, only male rats were included in this study, so sex-specific differences were not considered, which may limit the generalizability of the findings. Future studies with longer follow-up periods and deeper mechanistic investigations at cellular and molecular levels are warranted to better understand how biomolecular priming enhances stem cell-mediated regeneration and to more closely imitate the clinical progression of MRONJ in experimental models.

## Conclusions

The current findings reveal that priming hGMSCs with vitamins A and C at the defined pluripotency-inductive concentrations may enhance soft tissue integrity and promote osteogenic homeostasis, thereby contributing to the prevention and early treatment of MRONJ. This preventive strategy appears to sustain bone formation and mineral density, facilitating balanced bone remodeling through the upregulation of Runx2-mediated osteogenic differentiation. The present study’s results highlight the potential of combined vitamins A/C-based preconditioning as a prophylactic approach against MRONJ and offer new insights and strategies for reducing the risk of other bone-related complications.

## Supplementary Information

Below is the link to the electronic supplementary material.

Supplementary file1 (DOCX 30 KB)

## Data Availability

All data generated or analyzed during this study are included in this published article.

## Abbreviations

ALP: Alkaline phosphatase
ATRA: All-trans retinoic acid
BPs: Bisphosphonates
BMD: Bone mineral density
BMP: Bone morphogenic protein
DEXA: Dual-energy X-ray absorptiometry
hGMSCs: Human gingival mesenchymal stem/progenitor cells
MRONJ: Medication-related osteonecrosis of the jaw
MSCs: Mesenchymal stem/progenitor cells
OPN: Osteopontin
Runx2: Runt-related transcription factor 2
TGF-β1: Transforming growth factor- β1
ZA: Zoledronic acid

## References

[1] Aghaloo T, Hazboun R, Tetradis S. Pathophysiology of osteonecrosis of the jaws. Oral Maxillofac Surg Clin North Am. 2015;27:489–96. 10.1016/j.coms.2015.06.001. PMC4908822 26412796

[2] Kaibuchi N, Iwata T, Onizuka S, Yano K, Tsumanuma Y, Yamato M, et al. Allogeneic multipotent mesenchymal stromal cell sheet transplantation promotes healthy healing of wounds caused by zoledronate and dexamethasone in canine mandibular bones. Regen Ther. 2019;10:77–83. 10.1016/j.reth.2018.10.008. PMC6330512 30671501

[3] Fawzy El-Sayed KM, Dörfer CE. Gingival mesenchymal stem/progenitor cells: a unique tissue engineering gem. Stem Cells Int. 2016;2016:7154327. 10.1155/2016/7154327. PMC4903147 27313628

[4] Rady D, Abbass MMS, El-Rashidy AA, El Moshy S, Radwan IA, Dorfer CE, et al. Mesenchymal stem/progenitor cells: the prospect of human clinical translation. Stem Cells Int. 2020;2020:8837654. 10.1155/2020/8837654. PMC8063852 33953753

[5] Al-Qadhi G, Soliman M, Abou-Shady I, Rashed L. Gingival mesenchymal stem cells as an alternative source to bone marrow mesenchymal stem cells in regeneration of bone defects: in vivo study. Tissue Cell. 2020;63:101325. 10.1016/j.tice.2019.101325. 32223954

[6] Zhou LL, Liu W, Wu YM, Sun WL, Dorfer CE, Fawzy El-Sayed KM. Oral mesenchymal stem/progenitor cells: the immunomodulatory masters. Stem Cells Int. 2020;2020:1327405. 10.1155/2020/1327405. PMC7060886 32184830

[7] Fawzy El-Sayed KM, Mekhemar MK, Beck-Broichsitter BE, Bähr T, Hegab M, Receveur J, et al. Periodontal regeneration employing gingival margin-derived stem/progenitor cells in conjunction with IL-1ra-hydrogel synthetic extracellular matrix. J Clin Periodontol. 2015;42:448–57. 10.1111/jcpe.12401. 25875208

[8] Pizzicannella J, Marconi GD, Pierdomenico SD, Cavalcanti M, Diomede F, Trubiani O. Bovine pericardium membrane, gingival stem cells, and ascorbic acid: a novel team in regenerative medicine. Eur J Histochem. 2019;63(3):3064. 10.4081/ejh.2019.3064. PMC6767323 31696691

[9] Deshpande D, Karikal A, Kumar C, Kumar BM, Shetty V. In vitro evaluation of human demineralised teeth matrix on osteogenic differentiation of gingival mesenchymal stem cells. Arch Orofac Sci. 2022;17(2):247–58. 10.21315/aos2022.1702.OA08.

[10] Fawzy El-Sayed KM, Cosgarea R, Sculean A, Doerfer C. Can vitamins improve periodontal wound healing/regeneration? Periodontol 2000. 2023. 10.1111/prd.12513. 37592831

[11] Sherif MA, Anter E, Graetz C, El-Sayed KF. Injectable platelet-rich fibrin with vitamin C as an adjunct to non-surgical periodontal therapy in the treatment of stage-II periodontitis: a randomized controlled clinical trial. BMC Oral Health. 2025;25:772. 10.1186/s12903-025-06115-x. PMC12100897 40410805

[12] Elbehwashy MT, Hosny MM, Elfana A, Nawar A, Fawzy El-Sayed K. Clinical and radiographic effects of ascorbic acid-augmented platelet-rich fibrin versus platelet-rich fibrin alone in intra-osseous defects of stage-III periodontitis patients: a randomized controlled clinical trial. Clin Oral Investig. 2021;25:6309–19. 10.1007/s00784-021-03929-1. PMC8531044 33842996

[13] Wang Y, Singh A, Xu P, Pindrus MA, Blasioli DJ, Kaplan DL. Expansion and osteogenic differentiation of bone marrow-derived mesenchymal stem cells on a vitamin C functionalized polymer. Biomaterials. 2006;27:3265–73. 10.1016/j.biomaterials.2006.01.036. 16494940

[14] Fawzy El-Sayed KM, Nguyen N, Dörfer CE. Ascorbic acid, inflammatory cytokines (IL-1β/TNF-α/IFN-γ), or their combination’s effect on stemness, proliferation, and differentiation of gingival mesenchymal stem/progenitor cells. Stem Cells Int. 2020;2020:8897138. 10.1155/2020/8897138. PMC7448213 32879629

[15] Moore C, Fuentes C, Sauma D, Morales J, Bono MR, Rosemblatt M, et al. Retinoic acid generates regulatory T cells in experimental transplantation. Transplant Proc. 2011;43:2334–7. 10.1016/j.transproceed.2011.06.057. 21839265

[16] Fawzy El-Sayed KM, Hein D, Dorfer CE. Retinol/inflammation affect stemness and differentiation potential of gingival stem/progenitor cells via Wnt/β‐catenin. J Periodontal Res. 2019;54:413–23. 10.1111/jre.12643. 30830694

[17] Hassan A, Elbattawy W, Sanaa D, Nawwar A, Magdeldin S, Mokhtar M, et al. Retinol-augmented PRF versus PRF alone in periodontal regeneration: a randomized clinical trial. J Periodontal Res. 2025;60(8):847–9. 10.1111/jre.13404. PMC12476082 40400285

[18] Zhang S, Chen X, Hu Y, Wu J, Cao Q, Chen S, et al. All-trans retinoic acid modulates Wnt3A-induced osteogenic differentiation of mesenchymal stem cells via activating the PI3K/AKT/GSK3β signalling pathway. Mol Cell Endocrinol. 2016;422:243–53. 10.1016/j.mce.2015.12.018. 26747727

[19] Hore TA. Modulating epigenetic memory through vitamins and TET: implications for regenerative medicine and cancer treatment. Epigenomics. 2017;9:863–71. 10.2217/epi-2017-0021. 28554227

[20] Van Pham P, Tran NY, Phan NL, Vu NB, Phan NK. Vitamin C stimulates human gingival stem cell proliferation and expression of pluripotent markers. In Vitro Cell Dev Biol Anim. 2016;52:218–27. 10.1007/s11626-015-9963-2. 26487430

[21] Fawzy-El-Sayed K, Mekhemar M, Adam-Klages S, Kabelitz D, Dörfer C. TlR expression profile of human gingival margin-derived stem progenitor cells. Med Oral Patol Oral Cir Bucal. 2016;21:e30–8. 10.4317/medoral.20593. PMC4765758 26615501

[22] Soliman T, Ali Z, Zayed M, Sabry D, AbuBakr N. Assessing the bone-healing potential of bone marrow mesenchymal stem cells in jawbone osteoporosis in albino rats. Dent Med Probl. 2022;59:75–83. 10.17219/dmp/139200. 35254009

[23] Jin IG, Kim JH, Wu HG, Kim SK, Park Y, Hwang SJ. Effect of bone marrow-derived stem cells and bone morphogenetic protein-2 on treatment of osteoradionecrosis in a rat model. J Craniomaxillofac Surg. 2015;43:1478–86. 10.1016/j.jcms.2015.06.035. 26220883

[24] Huang J, Wang L, Tian W. Small extracellular vesicles derived from adipose tissue prevent bisphosphonate-related osteonecrosis of the jaw by promoting angiogenesis. Int J Nanomed. 2021;16:3161–72. 10.2147/ijn.s305361. PMC8114828 33994785

[25] Hokugo A, Christensen R, Chung EM, Sung EC, Felsenfeld AL, Sayre JW, et al. Increased prevalence of bisphosphonate-related osteonecrosis of the jaw with vitamin D deficiency in rats. J Bone Miner Res. 2010;25:1337–49. 10.1002/jbmr.23. PMC3153137 20200938

[26] Ansari S, Sarrion P, Hasani-Sadrabadi MM, Aghaloo T, Wu BM, Moshaverinia A. Regulation of the fate of dental-derived mesenchymal stem cells using engineered alginate-GelMA hydrogels. J Biomed Mater Res A. 2017;105:2957–67. 10.1002/jbm.a.36148. PMC5623163 28639378

[27] Alonso-Rodriguez E, González-Martín-Moro J, Cebrián-Carretero JL, Del Castillo JL, Pozo-Kreilinger JJ, Ruiz-Bravo E, et al. Bisphosphonate-related osteonecrosis. Application of adipose-derived stem cells in an experimental murine model. Med Oral Patol Oral Cir Bucal. 2019;24:e529-36. 10.4317/medoral.22959. PMC6667013 31232388

[28] AbuBakr N, Fares AE, Mostafa A, Farag DBE. Mesenchymal stem cells-derived microvesicles versus platelet-rich plasma in the treatment of monoiodoacetate-induced temporomandibular joint osteoarthritis in Albino rats. Heliyon. 2022;8:e10857. 10.1016/j.heliyon.2022.e10857. PMC9539788 36212013

[29] Farag DBE, Yousry C, Al-Mahallawi AM, El-Askary HI, Meselhy MR, AbuBakr N. The efficacy of *Origanum majorana* nanocubosomal systems in ameliorating submandibular salivary gland alterations in streptozotocin-induced diabetic rats. Drug Deliv. 2022;29:62–74. 10.1080/10717544.2021.2018522. PMC8725878 34964423

[30] Marx RE. Pamidronate (Aredia) and zoledronate (Zometa) induced avascular necrosis of the jaws: a growing epidemic. J Oral Maxillofac Surg. 2003;61:1115–7. 10.1016/s0278-2391(03)00720-1. 12966493

[31] On SW, Cho SW, Byun SH, Yang BE. Various therapeutic methods for the treatment of medication-related osteonecrosis of the jaw (MRONJ) and their limitations: a narrative review on new molecular and cellular therapeutic approaches. Antioxidants (Basel). 2021;5:680. 10.3390/antiox10050680. PMC8145192 33925361

[32] Beth-Tasdogan NH, Mayer B, Hussein H, Zolk O, Peter JU. Interventions for managing medication-related osteonecrosis of the jaw. Cochrane Database Syst Rev. 2022;7:Cd012432. 10.1002/14651858.CD012432.pub3. PMC9309005 35866376

[33] Kaibuchi N, Iwata T, Yamato M, Okano T, Ando T. Multipotent mesenchymal stromal cell sheet therapy for bisphosphonate-related osteonecrosis of the jaw in a rat model. Acta Biomater. 2016;42:400–10. 10.1016/j.actbio.2016.06.022. 27326918

[34] Nakagawa T, Tsuka S, Aonuma F, Nodai T, Munemasa T, Tamura A, et al. Effects of metformin on the prevention of bisphosphonate-related osteonecrosis of the jaw-like lesions in rats. J Prosthodont Res. 2021;65:219–24. 10.2186/jpr.JPOR_2019_629. 32938854

[35] Kushiro H, Takahashi H, Tanaka A. Effects of the prevention of medication-related osteonecrosis of the jaw by local administration of a dental pulp stem cell-conditioned medium to the rat tooth extraction socket. Odontology. 2021;109:836–44. 10.1007/s10266-021-00607-2. 33907964

[36] Yu X, Ge S, Chen S, Xu Q, Zhang J, Guo H, et al. Human gingiva-derived mesenchymal stromal cells contribute to periodontal regeneration in beagle dogs. Cells Tissues Organs. 2013;198:428–37. 10.1159/000360276. 24777155

[37] Fawzy El-Sayed KM, Bittner A, Schlicht K, Mekhemar M, Enthammer K, Hoppner M, et al. Ascorbic acid/retinol and/or inflammatory stimuli’s effect on proliferation/differentiation properties and transcriptomics of gingival stem/progenitor cells. Cells. 2021;10(12):3310. 10.3390/cells10123310. PMC8699152 34943818

[38] Kim D, Lee AE, Xu Q, Zhang Q, Le AD. Gingiva-derived mesenchymal stem cells: potential application in tissue engineering and regenerative medicine—a comprehensive review. Front Immunol. 2021;12:667221. 10.3389/fimmu.2021.667221. PMC8085523 33936109

[39] Poblano-Pérez LI, Castro-Manrreza ME, González-Alva P, Fajardo-Orduña GR, Montesinos JJ. Mesenchymal stromal cells derived from dental tissues: immunomodulatory properties and clinical potential. Int J Mol Sci. 2024. 10.3390/ijms25041986. PMC10888494 38396665

[40] Giles AJ, Hutchinson MND, Sonnemann HM, Jung J, Fecci PE, Ratnam NM, et al. Dexamethasone-induced immunosuppression: mechanisms and implications for immunotherapy. J Immunother Cancer. 2018;6:51. 10.1186/s40425-018-0371-5. PMC5996496 29891009

[41] Pizzicannella J, Diomede F, Gugliandolo A, Chiricosta L, Bramanti P, Merciaro I, et al. 3D printing PLA/gingival stem cells/ EVs upregulate miR-2861 and -210 during osteoangiogenesis commitment. Int J Mol Sci. 2019. 10.3390/ijms20133256. PMC6651609 31269731

[42] Cho SW, Sun HJ, Yang JY, Jung JY, Choi HJ, An JH, et al. Human adipose tissue-derived stromal cell therapy prevents bone loss in ovariectomized nude mouse. Tissue Eng Part A. 2012;18:1067–78. 10.1089/ten.TEA.2011.0355. 22220675

[43] Choi HK, Kim GJ, Yoo HS, Song DH, Chung KH, Lee KJ, et al. Vitamin C activates osteoblastogenesis and inhibits osteoclastogenesis via Wnt/β-catenin/ATF4 signaling pathways. Nutrients. 2019;11:506. 10.3390/nu11030506. PMC6471534 30818817

[44] Khojah Q, AlRumaihi S, AlRajeh G, Aburas A, AlOthman A, Ferwana M. Vitamin A and its dervatives effect on bone mineral density, a systematic review. J Fam Med Prim Care. 2021;10:4089–95. 10.4103/jfmpc.jfmpc_663_21. PMC8797105 35136772

[45] Abdik H, Avşar Abdik E, Demirci S, Doğan A, Turan D, Şahin F. The effects of bisphosphonates on osteonecrosis of jaw bone: a stem cell perspective. Mol Biol Rep. 2019;46:763–76. 10.1007/s11033-018-4532-x. 30506511

[46] Abu-Id MH, Warnke PH, Gottschalk J, Springer I, Wiltfang J, Acil Y, et al. Bis-phossy jaws"—high and low risk factors for bisphosphonate-induced osteonecrosis of the jaw. J Craniomaxillofac Surg. 2008;36:95–103. 10.1016/j.jcms.2007.06.008. 18234504

[47] Reid IR, Bolland MJ, Grey AB. Is bisphosphonate-associated osteonecrosis of the jaw caused by soft tissue toxicity? Bone. 2007;41:318–20. 10.1016/j.bone.2007.04.196. 17572168

[48] Xu HJ, Liu XZ, Yang L, Ning Y, Xu LL, Sun DM, et al. Runx2 overexpression promotes bone repair of osteonecrosis of the femoral head (ONFH). Mol Biol Rep. 2023;50:4769–79. 10.1007/s11033-023-08411-7. PMC10209270 37029290

[49] Chen J, He Y, Shan C, Pan Q, Li M, Xia D. Topical combined application of dexamethasone, vitamin C, and β-sodium glycerophosphate for healing the extraction socket in rabbits. Int J Oral Maxillofac Surg. 2015;44:1317–23. 10.1016/j.ijom.2015.06.011. 26149940

[50] Zhang W, Deng ZL, Chen L, Zuo GW, Luo Q, Shi Q, et al. Retinoic acids potentiate BMP9-induced osteogenic differentiation of mesenchymal progenitor cells. PLoS ONE. 2010;5:e11917. 10.1371/journal.pone.0011917. PMC2912873 20689834

[51] Xu QC, Wang ZG, Ji QX, Yu XB, Xu XY, Yuan CQ, et al. Systemically transplanted human gingiva-derived mesenchymal stem cells contributing to bone tissue regeneration. Int J Clin Exp Pathol. 2014;7:4922–9. PMC4152053 25197363

[52] Rodríguez-Lozano FJ, Oñate-Sánchez R, Gonzálvez-García M, Vallés-Bergadá M, Martínez CM, Revilla-Nuin B, et al. Allogeneic bone marrow mesenchymal stem cell transplantation in tooth extractions sites ameliorates the incidence of osteonecrotic jaw-like lesions in zoledronic acid-treated rats. J Clin Med. 2020;9(6):1649. 10.3390/jcm9061649. PMC7355877 32486396

[53] Zang X, He L, Zhao L, He Y, Xiao E, Zhang Y. Adipose-derived stem cells prevent the onset of bisphosphonate-related osteonecrosis of the jaw through transforming growth factor β-1-mediated gingival wound healing. Stem Cell Res Ther. 2019;10:169. 10.1186/s13287-019-1277-y. PMC6567445 31196208

[54] Mohammed BM, Fisher BJ, Kraskauskas D, Ward S, Wayne JS, Brophy DF, et al. Vitamin C promotes wound healing through novel pleiotropic mechanisms. Int Wound J. 2016;13:572–84. 10.1111/iwj.12484. PMC7949787 26290474

[55] Yuen DE, Stratford AF. Vitamin A activation of transforming growth factor-beta1 enhances porcine ileum wound healing in vitro. Pediatr Res. 2004;55:935–9. 10.1203/01.pdr.0000127023.22960.85. 15155862

[56] Diomede F, Marconi GD, Serroni M, Pizzicannella G, Trubiani O, Pizzicannella J. Ascorbic acid enhances bone parameter expression in human gingival mesenchymal stem cells. J Biol Regul Homeost Agents. 2019;33:1715–23. 10.23812/19-312-a. 31797649

[57] Valenti MT, Zanatta M, Donatelli L, Viviano G, Cavallini C, Scupoli MT, et al. Ascorbic acid induces either differentiation or apoptosis in MG-63 osteosarcoma lineage. Anticancer Res. 2014;34:1617–27. 24692690

[58] Escobar LM, Escobar JD, Bendahan Z, Castellanos JE. Retinoic and ascorbic acids induce osteoblast differentiation from human dental pulp mesenchymal stem cells. J Oral Biol Craniofac Res. 2021;11:143–8. 10.1016/j.jobcr.2021.01.002. PMC7840990 33537186

[59] Weng Z, Wang C, Zhang C, Xu J, Chai Y, Jia Y, et al. All-Trans retinoic acid promotes osteogenic differentiation and bone consolidation in a rat distraction osteogenesis model. Calcif Tissue Int. 2019;104:320–30. 10.1007/s00223-018-0501-6. 30635673

[60] Hore TA, von Meyenn F, Ravichandran M, Bachman M, Ficz G, Oxley D, et al. Retinol and ascorbate drive erasure of epigenetic memory and enhance reprogramming to naïve pluripotency by complementary mechanisms. Proc Natl Acad Sci USA. 2016;113:12202–7. 10.1073/pnas.1608679113. PMC5086989 27729528

[61] Hadzir SN, Ibrahim SN, Abdul Wahab RM, Zainol Abidin IZ, Senafi S, Ariffin ZZ, et al. Ascorbic acid induces osteoblast differentiation of human suspension mononuclear cells. Cytotherapy. 2014;16:674–82. 10.1016/j.jcyt.2013.07.013. 24176546

[62] Aghajanian P, Hall S, Wongworawat MD, Mohan S. The roles and mechanisms of actions of vitamin C in bone: new developments. J Bone Miner Res. 2015;30:1945–55. 10.1002/jbmr.2709. PMC4833003 26358868

[63] Henning P, Conaway HH, Lerner UH. Retinoid receptors in bone and their role in bone remodeling. Front Endocrinol (Lausanne). 2015;6:31. 10.3389/fendo.2015.00031. PMC4356160 25814978

