# Supplementary material for: Effect of vitamins A/C primed human gingival mesenchymal stem/progenitor cells in preventing medication-related osteonecrosis of extraction socket: an animal study
Source: Odontology. 2026 Jan 4;114(4):2214–25. doi: 10.1007/s10266-025-01288-x (PMC13630899; doi:10.1007/s10266-025-01288-x)
Supplement: Supplementary file 1 [file 10266_2025_1288_MOESM1_ESM.docx]

**Table S1.** Comparison of bone mineral density among all groups at each time point (2 and 4 weeks).

|  | Group I | | Group II | | Group III | | P value |
| --- | --- | --- | --- | --- | --- | --- | --- |
|  | Mean | SD | Mean | SD | Mean | SD |  |
| 2 weeks | 0.047 **^a^** | 0.015 | 0.138 **^b^** | 0.012 | 0.147 **^b^** | 0.009 | 0.0001* |
| 4 weeks | 0.065 **^a^** | 0.016 | 0.180 **^b^** | 0.014 | 0.204 **^c^** | 0.015 | 0.0001* |

*Significant difference at *P*<0.05 using ANOVA.

Means with different superscript letters are significantly different at *P*<0.05 using Tukey post hoc test.

n=5/time point.

**Table S2.**  Comparison of bone mineral density between 2 and 4 weeks within each group.

| Bone mineral density | | Mean | Standard Deviation | P value |
| --- | --- | --- | --- | --- |
| Group I | 2 weeks | 0.047 | 0.015 | 0.014* |
|  | 4 weeks | 0.065 | 0.016 |  |
| Group II | 2 weeks | 0.138 | 0.012 | 0.0001* |
|  | 4 weeks | 0.180 | 0.014 |  |
| Group III | 2 weeks | 0.147 | 0.009 | 0.0001* |
|  | 4 weeks | 0.204 | 0.015 |  |

*Significant difference at *P*<0.05 using paired t test. n=5/time point.

**Table S3.** Comparison of area percentage of newly formed bone among all groups at each time point (2 and 4 weeks).

|  | Group I | | Group II | | Group III | | P value |
| --- | --- | --- | --- | --- | --- | --- | --- |
|  | M | SD | M | SD | M | SD |  |
| 2 weeks | 0.20 **^a^** | 0.19 | 11.30 **^b^** | 0.56 | 23.00 **^c^** | 1.15 | 0.0001* |
| 4 weeks | 0.64 **^a^** | 0.06 | 26.80 **^b^** | 1.34 | 40.00 **^c^** | 2.00 | 0.0001* |

*Significant difference at *P*<0.05 using ANOVA.

Means with different superscript letters are significantly different at *P*<0.05 using Tukey post hoc test.

n=5/time point.

**Table S4.** Comparison of area percentage of newly formed bone between 2 and 4 weeks within each group.

| Area % of newly formed | | Mean | Standard Deviation | P value |
| --- | --- | --- | --- | --- |
| Group I | 2 weeks | 0.20 | 0.19 | 0.0001* |
|  | 4 weeks | 0.64 | 0.06 |  |
| Group II | 2 weeks | 11.30 | 0.56 | 0.0001* |
|  | 4 weeks | 26.80 | 1.34 |  |
| Group III | 2 weeks | 23.00 | 1.15 | 0.0001* |
|  | 4 weeks | 40.00 | 2.00 |  |

*Significant difference at *P*<0.05 using paired t test. n=5/time point.

**Table S5.** Comparison of relative gene expression of TGF-β1 among all groups at each time point (2 and 4 weeks).

|  | Group I | | Group II | | Group III | | P value |
| --- | --- | --- | --- | --- | --- | --- | --- |
|  | M | SD | M | SD | M | SD |  |
| 2 weeks | 1.00 **^a^** | 0.0 | 3.25 **^b^** | 0.16 | 3.80 **^c^** | 0.19 | 0.0001* |
| 4 weeks | 1.00 **^a^** | 0.0 | 4.50 **^b^** | 0.23 | 6.20 **^c^** | 0.31 | 0.0001* |

*Significant difference at *P*<0.05 using ANOVA.

Means with different superscript letters are significantly different at *P*<0.05 using Tukey post hoc test.

n=5/time point.

**Table S6.** Comparison of relative gene expression of TGF-β1 between 2 and 4 weeks within each group.

| Relative gene expression of TGF-β1 | | Mean | Standard Deviation | P value |
| --- | --- | --- | --- | --- |
| Group I | 2 weeks | 1.00 | 0.0 | ------- |
|  | 4 weeks | 1.00 | 0.0 |  |
| Group II | 2 weeks | 3.25 | 0.16 | 0.0001* |
|  | 4 weeks | 4.50 | 0.23 |  |
| Group III | 2 weeks | 3.80 | 0.19 | 0.0001* |
|  | 4 weeks | 6.20 | 0.31 |  |

*Significant difference at *P*<0.05 using paired t test. n=5/time point.

**Table S7.** Comparison of relative gene expression of Runx2 among all groups at each time point (2 and 4 weeks).

|  | Group I | | Group II | | Group III | | P value |
| --- | --- | --- | --- | --- | --- | --- | --- |
|  | M | SD | M | SD | M | SD |  |
| 2 weeks | 1.00 **^a^** | 0.0 | 3.50 **^b^** | 0.1769 | 5.40 **^c^** | 0.2693 | 0.0001* |
| 4 weeks | 1.00 **^a^** | 0.0 | 2.00 **^b^** | 0.0989 | 2.80 **^c^** | 0.1398 | 0.0001* |

*Significant difference at *P*<0.05 using ANOVA.

Means with different superscript letters are significantly different at *P*<0.05 using Tukey post hoc test.

n=5/time point.

**Table S8.** Comparison of relative gene expression of Runx2 between 2 and 4 weeks within each group.

| Relative gene expression of Runx2 | | Mean | Standard Deviation | P value |
| --- | --- | --- | --- | --- |
| Group I | 2 weeks | 1.00 | 0.0 | ------- |
|  | 4 weeks | 1.00 | 0.0 |  |
| Group II | 2 weeks | 3.50 | 0.1769 | 0.0001* |
|  | 4 weeks | 2.00 | 0.0989 |  |
| Group III | 2 weeks | 5.40 | 0.2693 | 0.0001* |
|  | 4 weeks | 2.80 | 0.1398 |  |

*Significant difference at *P*<0.05 using paired t test. n=5/time point.
